# How do dementia researchers view support tools for informed consent procedures of persons with dementia?

**DOI:** 10.1007/s00391-020-01779-2

**Published:** 2020-09-19

**Authors:** Theresa S. Wied, Aoife Poth, Johannes Pantel, Frank Oswald, Julia Haberstroh, ENSURE Consortium

**Affiliations:** 1 Psychological Aging Research (PAR), Faculty II—Education, Architecture, Arts, University of Siegen, Adolf-Reichwein-Str. 2a, 57068 Siegen, Germany; 2 Interdisciplinary Ageing Research (IAW), Goethe University Frankfurt, Frankfurt, Germany; 3 Institute of General Practice in Frankfurt am Main, Goethe University Frankfurt, Frankfurt, Germany

**Keywords:** Dementia research, Informed consent, Mental capacity, Supported decision-making, Demenzforschung, Informierte Einwilligung, Geistige Leistungsfähigkeit, Entscheidungsassistenz

## Abstract

The study aimed to assess how dementia researchers view eight support tools that have been defined to enhance informed consent (IC) procedures for people with dementia (PwD). In an online survey, 19 dementia researchers from Germany and Portugal evaluated the tools in terms of 4 implementation criteria. Overall, they all had a very positive attitude towards the support tools, whereby the tools person-centered attitude of the researcher and elaborated plain language were the most highly rated of the eight tools. Our findings also indicated that familiar support tools were assessed more favorably than those that were previously unknown. Overall, the results of this study showed that the participating dementia researchers were open to the use of decision support measures in PwD and were willing to apply the support tools in practice.

## Abstract (de)

Diese Studie zielte darauf ab, die Meinungen von Demenzforscher:innen zu 8 Entscheidungsassistenz-Tools zu erfragen, die zur Verbesserung der informierten Einwilligung für Menschen mit Demenz (MmD) definiert wurden. In einer Onlineumfrage bewerteten 19 Demenzforscher:innen aus Deutschland und Portugal die Tools hinsichtlich 4 Implementationskriterien. Insgesamt zeigten alle Befragten eine sehr positive Einstellung gegenüber den Entscheidungsassistenz-Tools. Die Tools „person-centered attitude of the researcher“ und „elaborated plain language“ wurden am positivsten bewertet. Die Ergebnisse deuten darauf hin, dass bereits bekannte Entscheidungsassistenz-Tools positiver bewertet werden als solche, die bislang unbekannt waren. Insgesamt zeigten die Ergebnisse dieser Studie, dass die teilnehmenden Demenzforscher:innen offen waren für die Nutzung von Entscheidungsassistenz-Tools und bereit waren, diese in ihrer eigenen Forschungspraxis einzusetzen.

## Introduction and background

Informed consent (IC) has to be obtained before patients can receive medical treatment or participate in research. Such IC has to meet three criteria: 1) a person with decision-making capacity 2) makes a free choice 3) following adequate information disclosure [1]. In order to be found competent, certain abilities, which can be impaired in persons with dementia (PwD), are required: understanding, appreciation, reasoning, and expression of a choice [1].

To assess whether the criteria required for IC are met, the focus is generally placed on the cognitive deficits and abilities that are impaired in PwD. As this approach does not correspond to the right to autonomy and equal legal capacity granted to PwD under the UN Convention on the Rights of Persons with Disabilities (UN-CRPD, in particular Article 12), it often ends in PwD’s having their autonomy taken away [3]; however, efforts to use a resource-oriented approach to support and treat PwD exist [2–5]. This approach attempts to identify the deficits and resources of PwD and to focus on remaining abilities in order to enhance their communication and decision-making abilities. A systematic review of supported decision-making in PwD [10] demonstrated that it is neglected in treatment and research decisions, and only rarely applied in care and everyday contexts. Tools to enhance the informed consent process for PwD, so-called enhanced consent procedures [6], have yet to be evaluated and implemented.

The present study was conducted as part of the transnational ENSURE project (*En*hancing the Informed Consent Process: *Su*pported decision-making and capacity assessment in clinical dementia *re*search), which, among other goals has defined, implemented and evaluated tools to improve IC for PwD. In the following, by the term ‘tools’ we mean both specific techniques and general approaches. On the one hand, tools include concrete strategies that should help a decision-making assistant to provide support. On the other hand, they include an attitude that appears necessary to provide decision-making assistance. In the course of the project, the following eight support tools were defined and pilot-tested: (1) Room Design Optimization, (2) Standardized Interview Structure, (3) Elaborated Plain Language, (4) Person-Centered Attitude of the Researcher, (5) Keyword List, (6) Priority Cards, (7) Visualization, and (8) Enhanced Written Consent Form [11]. These tools were defined in an expert consensus process of the ENSURE Consortium based on existing approaches on the support of PwD that were detected through a systematic literature search that is described in detail elsewhere [12]. After their content and structure had been critically discussed and adjusted to meet ethical and practical requirements, they were pilot tested and subsequently modified for improvement. For more detail on this process see Wied et al. [12]. In the study at hand, the tools were evaluated from two perspectives, firstly from the perspective of PwD, as assessed through qualitative interviews [12], and secondly from the point of view of dementia researchers. The present study addresses the latter.

### Aim and research question

The main aim of this pilot study was to find out what practitioners, in this case dementia researchers, think of the eight tools that were defined and implemented as part of the ENSURE project, and whether they would actually use them when conducting ICs with PwD. To achieve this aim, it was necessary to make use of implementation research, as this method examines the discrepancy between the theoretical development and realization of a concept [7]. According to Petermann [7], eight outcome parameters should be used to assess implementation: acceptability, adoption, appropriateness, feasibility, fidelity, cost, penetration, and sustainability. For the study at hand, it was only possible to assess four of them, as the others are factors that can only be measured following implementation. Our research question was therefore:

- how do dementia researchers evaluate the eight defined tools in terms of acceptability, appropriateness, feasibility, and adoption?

## Study design and investigation methods

### Participants

With support of the speakers and group leaders of the German Center for Neurodegenerative Diseases (DZNE) Witten and Greifswald dementia researchers were contacted via the DZNE mailing list. The inclusion criterion was that they had previously conducted an ICP, irrespective of whether they had previous experience with supported decision-making.

A total of 19 dementia researchers completed the online questionnaire. Their average age was 38 years (*SD* = 2.53 years) ranging from 24 to 57 years. Demographic data of the sample are displayed in Table 1. Most of them were research assistants (26.3%) or post docs (36.8%) and had completed their last ICP within the last 12 months (68.4%). Of the 19 researchers that completed the questionnaire, 17 were from Germany, and the remaining 2 from Portugal.

**Table 1:** Demographic data of the sample of dementia researchers (*N* = 19)

Background variables	Response options	*N*	M ± (range) or %
*Gender*	Male	7	36.8%
	Female	12	63.2%
*Age*		19	38.0 ± 2.53 (24–57)
*Country*	Germany	17	89.5%
	Portugal	2	10.5%
*Status*	Research assistant	5	26.3%
	Post doc	7	36.8%
	Professor	3	15.8%
	Other	4	21.1%
*Last conducted ICP*	Within the last 12 months	13	68.4%
	Within the last 4 years	19	100%
*Number of ICP in total*	1–10	7	36.8%
	11–20	3	15.8%
	21–99	4	21.1%
	>100	5	26.3%

### Procedure

We used UniPark by EFS Survey to develop an online questionnaire and distributed it to dementia researchers through email lists of the DZNE. The online questionnaire took the participants about 15 min to complete and was divided into three parts: (A) demographic questions, including requests for information on previous experience with ICs, (B) descriptions of the eight support tools, followed by an immediate evaluation of each tool and a final ranking, and (C) optional open-ended questions to explore driving and restraining forces for the use of supportive measures, and ask about other supportive measures they might know.

In the demographic part (A), the researchers were also asked how often they had conducted informed consent procedures (ICPs) and when the most recent one had taken place. In the second part (B), the eight tools were described and evaluated: (1) room design optimization involves the provision of adequate surroundings for the interview, including the avoidance of disturbances, restrictions in the number of attendees, tidiness and natural lighting. (2) Standardized interview structure describes the requirement that a structured conversation takes place, as well as stressing the importance of encouraging PwD to ask questions after each information segment, while their decision-making abilities, i.e. understanding, appreciation, reasoning and ability to express a choice, are simultaneously assessed. (3) Using elaborated plain language means using short and simple sentence structures (reduced syntactical complexity), connecting sentences with each other (semantic elaboration), speaking at a neutral pitch and normal speed (neutral prosody), and the avoidance of technical terms. (4) Person-centered attitude of the researcher means treating the PwD as equal partners in the decision-making process, taking their opinions and concerns seriously, and considering relationship aspects of communication by, for example, addressing them by name and maintaining eye contact. (5) A keyword list can be used to summarize the most important information after it has been provided verbally. (6) Priority cards should serve as an aid to help PwD weigh up risks and benefits, consider the consequences in their situation and everyday life, and to draw conclusions and explore reasons for their decision. (7) Visualization means illustrating important information to help reduce verbal memory load and simplify the process. (8) An enhanced written consent form should be prepared using elaborated plain language. It should be kept short and contain illustrations that use the same structure as the interview, where possible.

Each tool description was followed by a questionnaire that addressed the four dimensions of implementation [7] that were of interest: acceptability, appropriateness, feasibility and adoption*.*

Acceptability is the opinion reflected in a general or initial appraisal [9], and the extent to which someone considers an intervention agreeable or satisfactory [8]. In the case of the support tools, Acceptability was understood to describe a welcoming attitude (“I would welcome the use of [tool]”).

Appropriateness is defined as the “perceived fit” [9] of an intervention. For the support tools, this describes the suitability of the tool in the specific context of research with PwD (“[tool] seems fitting for …”). To increase specificity, the question on appropriateness was divided into two questions, one addressing appropriateness for the target group (“[tool] seems fitting for use in informed consent procedures with PwD”) and the other addressing appropriateness in a research setting (“[tool] seems fitting for use in informed consent procedures with PwD in research”).

Feasibility refers to practicability, and whether the intervention would be easy to carry out successfully [9]. This also includes the availability of the necessary resources [7] (“The use of [tool] seems to me to be realistic in terms of …”). The question on feasibility was also extended to raise specificity by asking researchers if they considered the tool to be realistic in terms of time required, qualification of the personnel, and the room situation.

Adoption is the intention or decision to use the intervention [7] (“I intend to use [tool]”).

Consequently, for each tool seven items were used to evaluate implementation criteria. Reliability for these was high, with Cronbach’s alpha ranging between 0.80 and 0.90 depending on the tool.

**Fig. 1:**
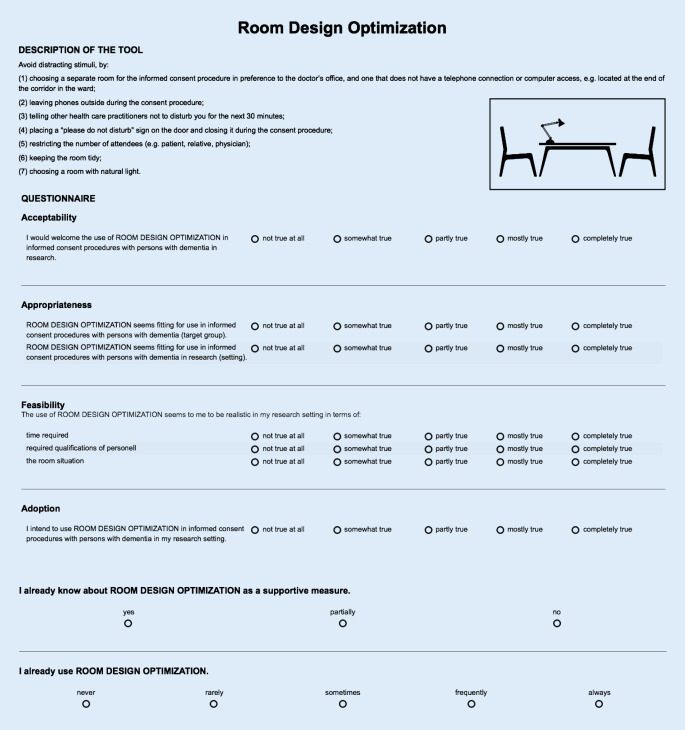
Exemplary tool description and evaluation questionnaire

A five-point Likert scale was used (1 = not true at all to 5 = completely true). The items were chosen and constructed in accordance with items tested by Weiner et al. [9]. Only few of the items which validity analysis showed to be useful [9] were finally chosen in order to keep the questionnaire as short as possible. At the end of the questionnaires on each tool, participants were also asked if they already knew (yes/partly/no) and/or used (1 = never to 5 = always) the tool. We also created a pictogram for each tool for visualization purposes and to simplify recognition. An example of a tool description and the corresponding questionnaire is depicted in Fig. 1.

**Fig. 2:**
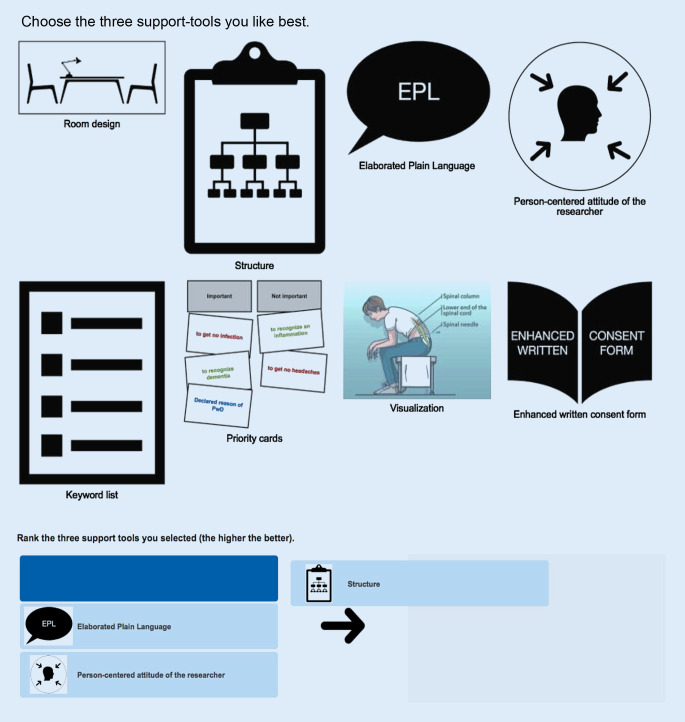
Pictograms of the eight described support tools. Participants were asked to choose the three that they like best, and then rank them

After evaluating each tool, participants were asked to choose the three tools they liked best, and then to rank those they had selected. This is depicted in Fig. 2.

In the final part (C), participants were asked to describe the driving and restraining forces for the use of the supportive measures and to describe other supportive measures they know of. A free-text field was also provided for them to make additional comments.

### Ethics

All procedures performed in the study were in accordance with the 1964 Helsinki declaration and its later amendments or comparable ethical standards. The study was reviewed by the institutional ethics committee of the University Hospital Frankfurt, which issued ethical approval in July 2017 (approval number E 92/17). Prior to completing the online questionnaire, participants received written information on the study and were then asked to consent to participate by clicking on a consent box.

### Descriptive analysis

We exported the gathered data from the online questionnaire platform UniPark and entered them into the Statistical Package for the Social Sciences (SPSS). For all data analyses we used SPSS Version 25.0 (IBM Corp, Armonk, NY, USA). After renaming and unifying variables, we created tables and charts to detect structures and correlations exploratively.

## Results

**Fig. 3:**
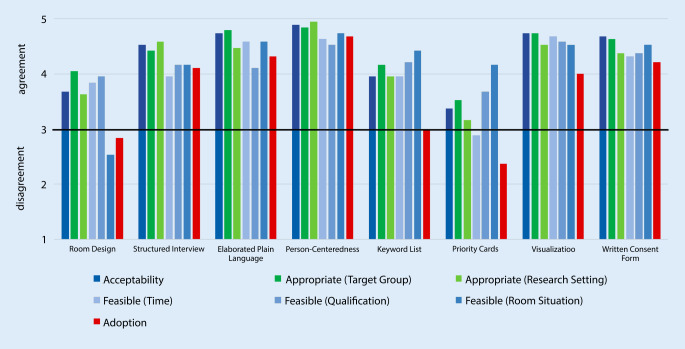
Evaluation of outcomes for the eight support tools (*N* = 19)

As can be seen in Table 1, most of the dementia researchers had conducted their last ICP during the last 12 months. Almost half of the participants had plenty of experience in conducting ICPs (47.4% had conducted more than 20, and 26.3% more than 100). The results of the researcher’s evaluation of the eight support tools are displayed in Fig. 3. Values of three and above (on or above the line) are considered to show agreement, while values below three are seen as disagreement. As can be seen, six out of eight tools received an overall rating of three and above, indicating agreement. The two exceptions are room design optimization and priority cards.

**Fig. 4:**
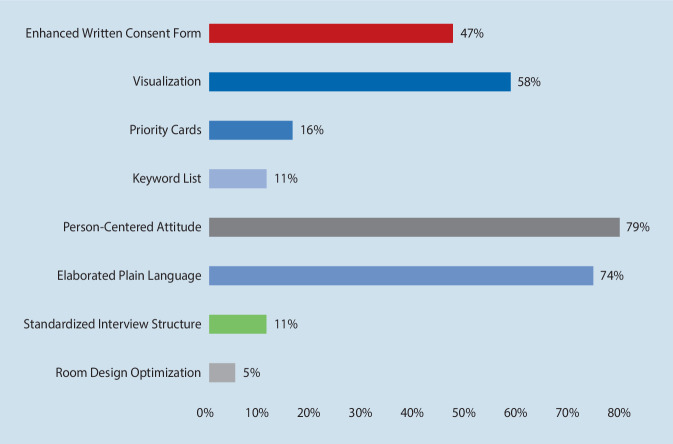
Percentages to which a tool was rated among the three chosen favorites (*N* = 19)

Figure 4 shows how often a tool was among the three chosen favorites in percent. The most popular tools were person-centered attitude of the researcher and elaborated plain language, followed by visualization and the enhanced written consent form.

Table 2 shows whether the researchers already knew the tool. It can be seen that person-centered attitude of the researcher and visualization were the best-known tools, whereas room design optimization, keyword list and priority cards were less familiar. Table 2 also indicates that compared with the mean ranking and the final evaluation score, the least known tools were rated worst, and vice versa. The final evaluation score reflects the tools’ placement in the final ranking of the researchers’ three favorite tools, whereby three points were assigned for being ranked first by a researcher, two for being ranked second and one for being ranked third, making the maximum amount possible for each tool 57 points (*N* = 19 × 3 points). The final evaluation is displayed in Table 2. As can be seen, the person-centered attitude of the researcher proved to be the clear winner. The second rank was taken by elaborated plain language, the third by visualization.

**Table 2:** Responses to the question whether the researchers already knew and used the tool, compared with the mean rating and the final evaluation score for each tool. Final evaluation score = sum of points assigned to each tool, 1st rank = 3 points, 2nd rank = 2 points, 3rd rank = 1 point. (*N* = 19)

Support tool	“I already know this tool”		“I already use this tool”		Mean rating of the implementation criteria (max. 5) ± SD	Final evaluation score (max. 57 points)
*(1) Room Design Optimization*	Yes	1	Always	0	3.50 ± 0.96	1
	Partially	6	Frequently	2		
	No	12	Sometimes	4		
			Rarely	3		
			Never	10		
*(2) Standardized Interview Structure*	Yes	6	Always	2	4.27 ± 0.61	2
	Partially	6	Frequently	5		
	No	7	Sometimes	4		
			Rarely	2		
			Never	6		
*(3) Elaborated Plain Language*	Yes	5	Always	1	4.51 ± 0.50	32
	Partially	10	Frequently	7		
	No	4	Sometimes	7		
			Rarely	1		
			Never	3		
*(4) Person-Centered Attitude of the Researcher*	Yes	10	Always	11	4.75 ± 0.42	39
	Partially	7	Frequently	3		
	No	2	Sometimes	3		
			Rarely	1		
			Never	1		
*(5) Keyword List*	Yes	1	Always	0	3.95 ± 0.71	5
	Partially	7	Frequently	2		
	No	11	Sometimes	2		
			Rarely	2		
			Never	13		
*(6) Priority Cards*	Yes	2	Always	0	3.31 ± 0.96	4
	Partially	3	Frequently	1		
	No	14	Sometimes	0		
			Rarely	1		
			Never	17		
*(7) Visualization*	Yes	10	Always	1	4.54 ± 0.51	19
	Partially	3	Frequently	2		
	No	6	Sometimes	6		
			Rarely	2		
			Never	8		
*(8) Enhanced Written Consent Form*	Yes	8	Always	3	4.44 ± 0.67	12
	Partially	8	Frequently	4		
	No	3	Sometimes	2		
			Rarely	2		
			Never	8		

## Discussion

The aim of the present study was to examine how dementia researchers would evaluate eight tools defined as part of the transnational ENSURE project to support decision-making of PwD during ICPs. The leading research question was: How do dementia researchers evaluate the eight defined tools in terms of acceptability, appropriateness, feasibility, and adoption?

The findings of the present study show that the overall attitude of the participating dementia researchers towards the eight defined support tools was positive. The tools standardized interview structure, elaborated plain language, person-centered attitude of the researcher, keyword lists, visualization, and enhanced written consent form were rated three and higher on all implementation characteristics, implying that they found the researchers’ approval. Furthermore, the majority of outcomes were assessed at four (“mostly true”) or above (“completely true”). This means that at the very least, the participating dementia researchers regarded these tools as mostly acceptable, appropriate, and feasible, and that they generally intended to use them in the future. This positive result shows that the researchers see a need for specific decision support measures that they are willing to apply. Even though researchers are willing to support the decision of PwD to participate or not in research, current research literature shows that knowledge about decision assistance, training for practitioners in the use of decision assistance and concrete decision support measures are lacking. Further research, but also the practical implementation of preliminary research results, is therefore urgently needed.

It is also worth taking a closer look at 1. room design optimization and 2. priority cards, the only tools whose implementation criteria did not find general approval (Fig. 3). 1. Room design optimization was not rated as feasible in terms of the room situation and the participating researchers did not intend to use it in the future (adoption was low). This may reflect a lack of suitable rooms, or rooms that could be optimized in accordance with our recommendations, at their research institutions. 2. Priority cards were considered too time-consuming to be feasible. Nevertheless, they were rated feasible in terms of qualification of personnel and the room situation. Adoption was also rated low for priority cards, which means the participants do not intend to use them. It would appear that the use of priority cards is regarded as too complex in a research context, but not a bad approach in general. It may therefore make sense to test them in a different context.

Another interesting result is that researchers evaluated the tools they already knew more highly, while those that were less well known were less popular and received lower scores. This may mean that researchers are more open to decision support measures they already know, and that they are more likely to integrate them into their own research practice. One reason for this is perhaps that researchers, whose time is already limited, may assume that the implementation of new tools would involve extra work.

Furthermore, the very positive rating of elaborated plain language (EPL) is interesting and worth a closer look. On the one hand, the researchers gave EPL their strong agreement in terms of all four implementation outcomes and, unlike the priority cards, had no concerns about the time required to use this tool. On the other hand, most researchers said that they knew about EPL to some extent and used it either occasionally or frequently. Since EPL is a very complex form of spoken language, the use of which requires both considerable time and practice, and training courses in its use are not currently available, we suspect that the researchers equated or confused EPL with the use of simplified language and the avoidance of technical terms.

### Limitations

The sample size of the present study was naturally restricted by the low number of dementia researchers in general, but nevertheless, small. Recruitment, which was originally planned to take place in the three countries participating in the ENSURE project (Germany, Spain and Portugal), turned out to be rather difficult, and the sample ended up being unevenly composed. As 17 of the dementia researchers were German and 2 Portuguese, the results can only be generalized to a limited degree. Unfortunately, we also did not have access to information about the response rate to our email.

Another limitation is that most of the participants were not principal investigators (PI) and hence do not have control and authority to all informed consent approaches; however, we think that all researchers are responsible for the informed consent they perform themselves, and have, at least to some degree, the possibility to shape the support provided. Nevertheless, the high popularity of EPL might be explained by the low number of PI’s in our study sample, as it is a tool that is applicable without any institutional authority, in comparison to room design optimization*,* which showed low popularity as it is a tool that has to be authorized and implemented by the institution. Unfortunately, we do not know why the participation of PI’s was so low.

Furthermore, the sample of dementia researchers was highly selective. All of them had an open-minded attitude towards the defined tools and our research in general, and they gave helpful feedback by providing detailed answers in the free-text fields at the end of the questionnaire. Due to the small sample size, only a descriptive analysis of these data was possible.

We acknowledge that the techniques and approaches that we refer to as ‘tools’ differ considerably in their nature and complexity, and that consequently comparability of their ratings may be limited. Moreover, although our instrument of measurement was based on existing measures of implementation criteria [7, 9], it had not yet been pilot tested in the form used.

Finally, the present study did not take the psychopathology of dementia into account, which should also be considered when developing and implementing support tools. As it is a progressive neurodegenerative disease, people with dementia are very likely to reach a stage at which the tools can no longer unfold their supportive effect. The aim should nevertheless be to maintain autonomy as long as possible.

## Conclusion and/or practical recommendations

This study provided a preliminary insight into researchers’ views on the extent to which specific decision support measures could be implemented and integrated into their daily research routines. The study also provides an initial overview of the willingness of researchers to use such tools to support decision-making in PwD.

Overall, the results of this study showed that the participating dementia researchers were open to the use of decision support measures in PwD and were willing to apply the support tools in practice. This result shows the importance of developing training courses in the field. Such training courses should communicate information on innovative decision support measures, and enable participants to practice the application of such tools. Through these training offers, the openness to still unknown support measures might be further promoted. In consideration of the ethical and legal requirements declared in the UN-CRPD described above, the urgency of providing information and training for decision support tools is evident.
